# Cure of Aneurism by Digital Compression

**Published:** 1859-08

**Authors:** 


### EDITORIAL DEPARTMENT.

*Cure of Aneurism by Digital Compression.*—The January No. of the "*North American Medico-Chirurgical Review*" contains an admirable article from the pen of Dr. S. W. Gross, on the treatment of aneurism by digital compression. This is by no means a novel procedure; but the case reported by Dr. Gross is one of the most perfect examples possible of the value of such a measure.

The patient was a negress, thirty-two years of age, with an aneurismal tumor, 4½ inches long and 5½ inches wide, situated in Scarpa's triangle, about an inch and a half below Poupart's ligament, on the right thigh. She had complained of shooting pains in the limb, &c., for about ten months, and the tumor had been noticed for about two months, commencing with a hard lump "about the size of a walnut without its hull;" *bruit*, pulsation, and all the signs of aneurism, were well marked, and the sac could readily be emptied.

Dr. Gross, having provided a suitable number of intelligent assistants, commenced the treatment June 10th, at ten minutes past ten A. M. A grain of morphia was given at the commencement, and a half-grain at one P. M. Pressure was kept up, the assistants relieving each other for 31¼ hours; a grain of morphia was then administered, and pounded ice in a bladder was applied, to be continued throughout the night. This was on Friday, at 5½ P. M., when the tumor was very hard, not diminished in size, with no thrill, no *bruit*, and very feeble pulsation. On Saturday, at twenty minutes before eleven A. M., the compression was renewed, and continued for fourteen hours and thirty-five minutes. Dr. Gross further remarks that the compression was such as to almost entirely arrest circulation in the tumor, and he is confident that had the compression been continued for two or three hours longer at the first sitting, a cure would have been the result. This was prevented on account of the pain to the patient and the fatigue of himself and his assistants, confident that the next sitting would be successful. On the thirteenth of October the patient came to the city to report herself. "The tumor had diminished to the size of a walnut, and was very solid. The locomotion was perfect, and the health good in every respect." This was about four months after the commencement of the treatment.

Dr. Gross has collected and tabulated cases of aneurism treated by this method, from the first cases treated in 1844 by M. E. Greatrez, up to the date of his own case, making in all twenty-three cases. "The number of the different varieties and their results are shown in the following table:—

| *Variety.* | *No. of Cases.* | *Cure.* | *Failure.* |
|---|---|---|---|
| Popliteal - - - | 15 | 10 | 5 |
| Femoral - - - - | 4 | 3 | 1 |
| Inguinal - - - - | 2 | 0 | 2 |
| Arterio-Venous - - - | 2 | 2 | 0 |
| | 23 | 15 | 8 |

After a review of the cases which he has collected, the author closes with the following propositions:—

"I. Digital compression, uncombined with apparatus, was first attended with success in the hands of Dr. Knight; but to M. Vansetti is due the merit of having first introduced it into practice.*

"II. It has never been followed by bad consequences; and when not successful, it so modifies the tumor and collateral circulation as to render a cure by other means almost certain.

"III. It has been employed alone, either previous or subsequent to mechanical compression, in fourteen instances, eight being failures.

"IV. In only seven cases has it been employed primarily and alone, and in all but two with perfect success.

"V. When double and alternating, it has effected cures in every case, five in number, and therefore deserves special attention.

"VI. In most of the cases the compression has been total; but this is not necessary for a favorable result.

"VII. It has effected cures, whether it was continued, interrupted, or intermittent; in some cases the patient applying the pressure.

"VIII. When properly employed and continued for a sufficient length of time, and the cases are suitable ones, it can scarcely fail to accomplish a cure. Inguinal aneurisms are not fit cases for this procedure.

"IX. It is less apt to give rise to inflammation in the integument, and has been borne where mechanical pressure has produced an eschar.

"X. It can be used when apparatus has failed or is intolerable. In a majority of these cases cures have been accomplished.

"XI. In certain situations it may be made to bear upon the artery alone. It is far less painful and requires a much shorter time for a cure, than any other method of treatment."

This is, in brief, a summary of the important points contained in Dr.

* Mr. Greathrez employed digital, in connection with mechanical compression. The fingers were but accessory to the instrument.

Gross' article, a communication which does infinite credit to his skill and perseverance. We had set it aside for a place in our eclectic department; but a press of matter has prevented its appearance until now, when we have considered it sufficient to present an abstract to our readers. We are rather ashamed to be behind our trans-Atlantic brethren in this matter; for an abstract of the article and a complimentary notice of the author has already appeared in the *Gazette Hebdomadaire.* This gives us an occasion, however, to congratulate our friend on the recognition of his labors abroad and the compliment of a translation into a foreign language.

